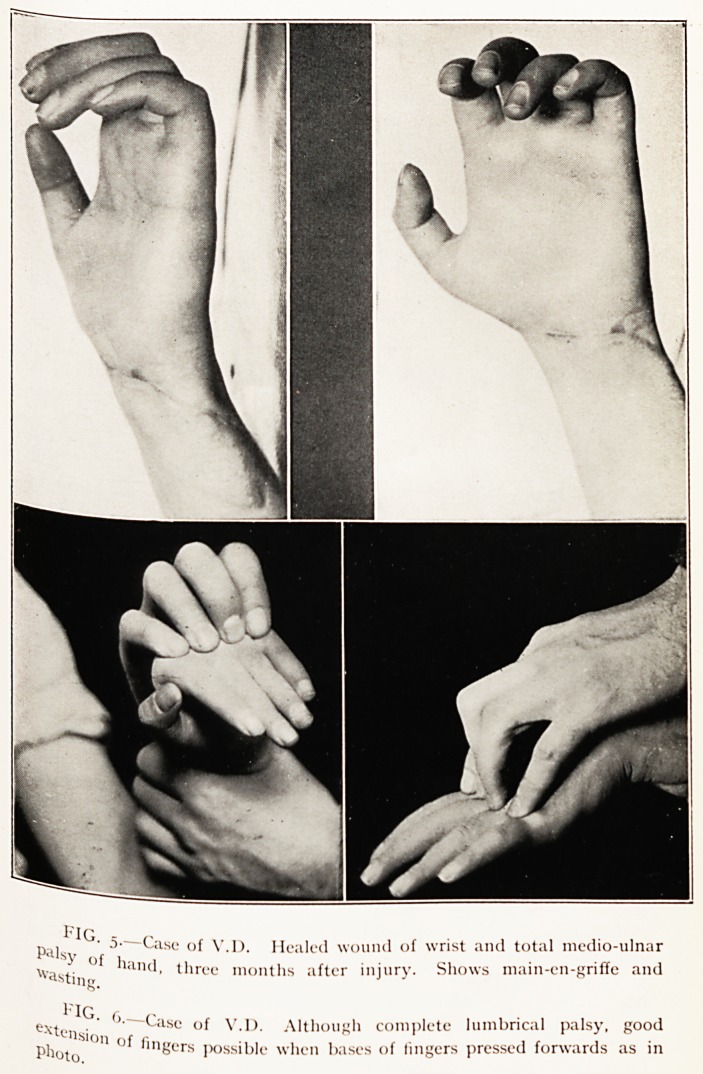# A Comparative Review of Spinal and Peripheral Nerve Injuries
1Case Demonstration at the meeting of this Society, November 12th, 1924.


**Published:** 1925-01

**Authors:** Wilfrid Adams

**Affiliations:** Assistant surgeon, Bristol Royal Infirmary; Surgeon to Out-patients, Bristol Royal Hospital for Sick Children and Women


### A COMPARATIVE REVIEW OF SPINAL AND PERIPHERAL NERVE INJURIES.[1]

(*With illustrative cases.*)

BY

WILFRID ADAMS, M.S., F.R.C.S.,

*Assistant Surgeon, Bristol Royal Infirmary;*
*Surgeon to Out-patients, Bristol Royal Hospital for Sick Children and Women.*

THE misfortunes under consideration are remarkable both in the dramatic effect of their onset and in the very slow process of their recovery. In them the task of diagnosis is often not an easy one, for nerve injury is apt to be disguised by marked bruising of the tissues and fracture or dislocation. During the restoration of function, which may take months or years, the unfailing co-operation of the patient must be maintained, often an irksome appliance has to be worn, and always hopes of recovery must be encouraged. From the patient's point of view, though it but rarely demands life itself, it frequently entails the temporary or permanent loss of use of a limb. Such features make the subject one of vital importance.

[1] Case Demonstration at the meeting of this Society, November 12th, 1924.

Included in this article are notes of part of the clinical material which inspired its composition. Among them features of singular interest are :—A case of medio-ulnar recovery with a gap, the precise localisation of compression in a case of delayed ulnar paralysis, and a note on the action of the lumbrical muscles.

*The Varieties of Nerve Injuries* may be classed as deliberate and accidental.

A. *Deliberate Injuries.*—The surgeon intentionally divides nerves in the following instances : (1) Posterior rhizotomy,

*Diagram showing sites for nerve division in Fœrster's* (F) *and Stoffel's* (S) *operations.*

*i.e.* division of the sensory roots of the spinal cord. The purpose of this (Fœrster's operation) is either to stop painful impulses from the nerves concerned passing to the brain (as in tabetic crises or intolerable neuritis), or to eliminate spasticity in muscles. The latter effect is due to the fact that section of the posterior root breaks the reflex arc, preventing sensory impulses reaching the spinal cord from the muscles concerned. On the integrity of this arc muscle tone depends in part. So the surgeon cuts post-nerve roots to eliminate the crippling spasm of muscles, like the adductors of the thighs in Little's disease (spastic diplegia). The analgesia or hypotonia required can be produced, and yet the use of essential muscle groups, such as the quadriceps extensor is preserved, if the series of roots divided is not quite continuous.

(2) The peripheral nerves are cut: (*a*) at amputations, (*b*) in resections of nerves for tumour or incurable neuritis, (*c*) in Stoffel's operation, *i.e.* division of the nerves to over-acting groups of muscles which has recently come into favour. This serves to restore muscle balance in a leg where there is adductor or equinus deformity resulting from spastic paralysis.

B. *Accidental Injuries.*—Cases fall into three groups. (1) During operations in the vicinity of nerves, such as the ulnar at the elbow, a nerve may be divided, ligated, or bruised. The onset of symptoms may be delayed. (2) As a result of a single severe accident nerves are apt to be divided by incised wounds chiefly near the wrist. The fractures in which nerves are involved most often are those of the humerus, while cases of delayed onset tend to follow injuries to nerves about the elbow. Commonly in civilian practice the nerve is only bruised or partly divided, often the onset of symptoms is delayed, and spontaneous recovery is frequent. (3) The result of repeated minor injuries or continued pressure, *e.g.* "crutch palsy" and "cervical rib."

*Symptoms* are organic and functional. In onset they may be immediate, delayed, or recurrent. In the last group symptoms occur at the time of the initial bruising. They pass off in a few days, but reappear later, owing to peri-neural fibrosis or ossification.

The symptoms vary according to the proportion of the different nerve elements present in any particular case. Thus with division of the musculo-spiral and 5th cervical nerve root there are marked motor but trifling sensory changes.

Loss of sensation in complete nerve division is accompanied by a notable absence of pain, whereas the reverse is often true in partial lesions. The comparatively small area with lost deep sensibility (pressure on tendons, bones, and joints) calls for explanation. When a nerve trunk is divided in the lower part of a limb sensory fibres to deep structures will escape, because they have already left the nerve high up and run down in the muscle substance and tendon.

The paralysed muscles wither, and are prone to over-stretching by unchecked antagonists. While after the tenth day the characteristic "reaction of degeneration" occurs, and trophic signs appear in the skin, and nutrition of bones and joints suffer.

Partial nerve lesions may show mainly paresis, or be associated with marked pain. In the case of median and sciatic nerves such injuries, when associated with excessive scar formation (as in war wounds), induce the very painful condition termed "causalgia," in which the dominant symptom is an intense burning pain (hence the name of the condition), which is relieved by immersion in water or rendered excruciating by pressure.

In connection with the diagnosis of nerve injuries the functional factor provides an unpleasant trap, as in the following instance. A collier was sent me by his doctor, suffering from marked varus deformity and pain in the right foot. It had persisted four weeks since a severe wrench of ankle. I diagnosed sprained external lateral ligament and treated him accordingly. At the end of three months, however, he relapsed, and the deformity was as bad as ever. Shortly after a surreptitious glance at him as he was dressing himself revealed the foot quite straight and ready to slide into his boot!

*Treatment.*—In order to estimate the progress of recovery it is necessary to map out the distribution of the paralysis at the outset. Treatment varies with the class of iujury.

A. *Open wound absent.*

Splintage to relax both nerve and palsied muscles. Warm covering and electro-therapy, and, if there is no recovery after six months, exploration is advisable. Callus may call for removal in cases of delayed onset.

B. *Open wound present.*

When the toilet of a wound is performed nerves in the neighbourhood should be investigated. If divided they are resutured, and in all cases they should be diverted along undamaged tissues if possible.

*Recovery.*—Sensation returns first, and is heralded by the appearance of Tinel's sign of "distal tingling on percussion." This depends on the fact that the advancing ends of regenerating fibres are tender for a time, during which, in common parlance, "life is returning to the part." If the surface-marking of the nerve is used as a guide and one percusses along its length, starting distally, when one reaches the level of the growing end of the nerve the patient will complain of a tingling pain referred to the area of distribution of the nerve. Feeling in the paralysed region begins to return about the third month, but for some weeks only an unnatural tingling occurs, no matter what form of stimulus (touch, pin-prick, or thermal) is applied.

Motion returns at about twelve to twenty-four months.

*Late Operation.*—If recovery fails to commence at the expected time, the safe rule is to advise operation, on the assumption that the nerve has been divided or compressed, and the ends are out of contact. Before intervening thus one must be sure that the nerve is capable of regeneration (ant. cornual cells atrophy after three years' disuse), and that the tissues to which the nerve is to regrow are supple and capable of responding to stimuli.

The ends of the nerve are identified with certainty by full exposure. They are pared down until healthy nerve bundles are seen, and then sutured with care to avoid tension and torsion. The need for the last precaution is based on the fact that fibres are not mixed indiscriminately in the nerve trunk, but segregated into bundles according to function. Just as various motor and sensory tracts can be plotted out on a transverse section of the spinal cord, so we are discovering that the fibres seen at the cut end of main nerves are arranged in groups according to varying functions. At once the risk during re-union of disturbing this relationship becomes obvious. For example, little use could result from joining, however neatly, a motor bundle, sending impulses downwards, with a sensory bundle. If such were done motor fibres destined for motor-end plates would reach sensory nerve endings in the skin! To this complication the frequent failures after suture of mixed nerves are due.

*Gap Cases.*—In some cases a gap is present between the divided ends, and how to bridge such has long been a vexed problem. Thanks to the ample experience in the recent war we are now certain that there is no efficient substitute for direct union of the severed ends, and that alternatives, such as nerve graftings, are valueless. The following means help to overcome the difficulty: (1) extensive freeing of the upper and lower ends; (2) two-stage suturing—after preliminary suture under tension the nerve may have stretched enough to allow satisfactory union some weeks later; (3) displacement—an inch is gained for the ulnar nerve if it is transferred from back to front of elbow; (4) resection of bone—accommodating the length of limb to suit the nerve.

*Prognosis.*—In a comprehensive review of late results of end-to-end suture of nerves injured in the recent war Platt and Bristowe (*Brit. Journ. of Surg.*, January, 1924) state " complete failures will be found in about twenty per cent.," and the " musculo-spiral heads the list of recoveries, and may be expected to show practically complete restoration of function in at least fifty per cent. of the successful cases."

*Hopeless Cases.*—If nerve regeneration fails useful movement may be obtained in certain cases by tendon transplantation, as in the successful conversion of flexors of wrist into extensors in cases of musculo-spiral palsy. Finally, some mechanical appliance, such as a splint or walking-iron, may be the best that can be done to help the cripple. Happily a permanent ulnar palsy is but a slight handicap, although the insensible weak index finger in median cases is very disabling.

*Traumatic Neuritis.*—Every case of this sort without obvious cause should be cautiously reviewed to exclude functional disease before advising operation.

One of the common examples is found after amputations where the nerves have been cut flush with the other tissues and are caught in scar or exposed to pressure at the end of the stump. This should be avoided by severing the nerves individually an inch or so higher than the bone, and must be remembered even where digital nerves are concerned, otherwise the risk of a second operation will be incurred.

Operative measures employed, in addition to local neurectomy, include the injection of alcohol (seventy per cent.) into the nerve proximal to the lesion, Leriche's operation of decortication of the sympathetic plexus from the main artery of the limb, and as a last and somewhat dangerous resort Fœrster's operation of section of the posterior spinal roots.

### Illustrative Cases shown at the Meeting.

**Case 1.**—*Baby H., aged 4 months. Birth palsy and recovery.* Suffered considerable trauma during a difficult birth eight days before she was brought on August 10th, 1924, when I found Erb's palsy (L), fractured shaft of humerus (R) and parietal cephalhæmatoma (L.)

When shown three months later she was fully recovered.

I have had similar cures with two other cases, in both of which there was a homolateral fracture of the humerus. The occurrence of these palsies depends on the fact that the roots of the brachial plexus are liable to stretching by vertical traction on the arm. The stress falls chiefly on the upper roots (Erb's palsy) if the pull is downward, or on the lower roots (Klumpke's palsy) if upward. In Erb's palsy the fifth cervical root mainly suffers, and the limb assumes a characteristic position described as that of "policeman awaiting a tip," *i.e.* with the palm looking upwards and backwards at the end of the dependent and inwardly rotated arm. This is due to paralysis of deltoid, external rotators of shoulders, flexors of elbow and extensors of wrist. The treatment used in all my cases was postural, the arm being kept raised with the hand against the nape of the neck. This relaxes the affected part of the plexus and the paralysed muscles. Birth palsy may be present without fracture. Good return of power is the rule, but is the less likely the more extensive the lesion. If permanent palsy remains in the upper root lesions the faulty muscle balance leads, after a year or two, to a characteristic attitude called "the stomach-ache position." In this case the head of the humerus is pressed to the back of the glenoid cavity and the pronated hand is carried across the abdomen. I have had one such case, aged eight years, which illustrated well the fact that, owing to contracture of the unantagonised muscles (*e.g.*, subscapularis) in front of the shoulder-joint, the limb, if rotated outwards passively and released, swings back briskly on to the abdomen again like a spring-door. In such late cases correction by tenotomy and capsulotomy may restore useful freedom to the limb.

**Case 2.**—*W.L., aged 49 years. Sub-total brachial palsy with good recovery.*

December 6th, 1923. A subcoracoid dislocation occurred at the left shoulder and was reduced. About a week later extensive paralysis of the arm ensued. Pain was slight and sensory loss was confined to the hypothenar eminence, but the whole limb hung limp and motionless by the side except for slight, ineffective movements of some muscles at the shoulder and elbow.

He was able to resume work in July, 1924, and on Nov. 12th, 1924, had full range of movement and good power.

**Case 3.**—*W.H., aged 7 years. Lesion of musculo-spiral nerve with compound fracture of the humerus. Partial recovery.*

November, 1922. A typical wrist drop followed shortly after a very severe motor-lorry accident, with compound fracture of the humerus. A duralumin wire cock-up wrist splint with thumb-ring was applied.

February 20th, 1923. A sequestrectomy was performed, since when the wound has healed, although with much scarring.

November 12th, 1924. Partial recovery has occurred, the extensors carpi radialis longus and brevis are efficient, while the extensors communis digitorum and longus pollicis are acting feebly.

There is a choice of further operative measures which may be used to perfect the partial recovery of power in the extensor muscles. Firstly, neurolysis of the musculo-spiral nerve; but this is of doubtful value owing to the long area of scarring around it. Secondly, active flexor tendons may be transplanted to reinforce the partially recovered extensors.

**Case 4.**—*B.H., aged 9 years. Triple lesion of the elbow, affecting musculo-spiral, median and ulnar nerves. All recovering.* (Figs. 1 and 2.)

May 29th, 1923. A severe supracondylar fracture of the humerus defied full reduction. Marked myositis occurred and, in the second week, lesions of the three nerve trunks about the elbow began to develop with the usual resultant deformities. Splintage, etc., were employed.

March, 1924. A trophic sore appeared on the dorsum of the little finger and deepened, despite treatment. A radiogram was taken to see if underlying bone or joint were infected, but in June, 1924, improvement set in and rapid healing. This was a pleasant token of ulnar nerve recovery. By November 12th, 1924, good recovery of the musculo-spiral was present. Restored function was present also in the medio-ulnar territory, but with an obvious and mystifying gap which calls for remark. While the flexors to the wrist and the intrinsic muscles of the hand were now active the intermediate long flexors of fingers and thumb remained withered and inert. Strikingly parallel was the sensory defect, for while wool and pin-point were appreciated by the patient, squeezing of the phalanges was not felt. It is hard to believe that the compressing force could have had a selective action of this destructive kind on the particular fibres of median and ulnar nerves concerned in the non-recovered parts. I am inclined to attribute the

PLATE II.

FIG. 1.

FIG. 2.

FIG. 1.—Case of B.H. Recovering triple nerve lesion of elbow eighteen months after injury.

FIG. 2.—Case of B.H. Skiagram of elbow, eighteen months after injury, showing unequal growth of humeral epiphysis.

PLATE III.

FIG. 3.—Case of M.M. Ulnar palsy of hand four months after injury at elbow.

FIG. 4.—Case of M.M. Shows callus and the notch where it compressed the ulnar nerve.

muscle, and so the deep sensory, implication of the digits to Volkmann's ischemic contracture of the forearm muscles. The latter suffered severe bruising and swelling from the accident and subsequent manipulations. During the maintenance of the acutely flexed position of the elbow for three weeks following they may have been unduly compressed against the front of the upper arm. When the recovering nerve fibres reached the contractured muscles their further growth would be inhibited by a condition of impermeable fibrosis.

**Case 5.**—*M.M., aged* 10 *years. Ulnar nerve lesion of delayed onset. Recovering.* (Figs. 3 and 4.)

July 11th, 1924. Injury to the lower epiphysis of the right humerus with much bruising. No neural lesion was present. Reduction was satisfactory. On August 3rd, 1924, symptoms of a complete ulnar nerve lesion were found. On October 1st, 1924, recovery was heralded by the appearance of. Tinel's sign in the forearm, and on November 12th, 1924, a typical hyperæsthesia to deep pressure and pin-prick with absent epicritic feeling showed sensation was returning.

Unusual interest centres round the radiogram of November, 1924. Just above the elbow-joint the ulnar nerve was to be felt running down behind an unusual bony boss. At a corresponding site in the radiogram is a piece of callus with a notch in it. This, I believe, lodges the ulnar nerve, and so marks the precise level and manner of compression of the nerve, symptoms of which commenced a month after the accident. Recovery is proceeding, and may reach the muscles shortly, so that neurolysis is hardly desirable at this early date.

Subsequent events have shown this course to be the wise one, for on January 25th, 1925, motor recovery was obvious and deformity gone.

**Case 6.**—*W.D., aged* 3½ *years. Division of ulnar nerve by incised wound above wrist. Delayed operation. Recovering.*

December, 1923. Right forearm cut by broken glass and stitched.

June, 1924. He was sent to me with a complete ulnar lesion and no signs of recovery. Operation revealed a markedly bulbous proximal end of the nerve, which was turned aside and out of contact with the scarred lower end. It was sutured in accordance with the rules already outlined, but sufficient time has not yet elapsed (November, 1924) for one to judge of the result. When seen (January, 1925) it was obviously recovering. Deformity gone and good Faradic response in interossei. This unusually rapid return of function depends doubtless on the tender age of the child.

**Case 7.**—*V.D., aged 20 years. Incised wound with division of median and ulnar nerves at wrist. Primary suture performed. Recovery commencing.* (Figs. 5 and 6.)

(I was indebted to Mr. Rendle Short for permission to show this case.)

July 5th, 1924. She put her hand through a glass pane, cutting the median and ulnar nerves, together with superficial tendons on the front of the wrist. Immediate suture was performed. November 12th, 1924. A typical hollow palm, concavity of the surfaces of the thenar and hypothenar eminences, main-en-griffe and delicate, pinkish skin were present.

Recovery was ushered in by the development of an unnatural tingling sensation when the centre of the palm was pressed, and by November 12th, 1924, this perverted response alone resulted all over the paralysed area no matter what variety of stimulus (*e.g.* wool, pin-pricks, thermal) was applied. In the affected muscles the galvanic response was still that of degeneration, but the abductor indicis alone was typical among them in giving the A.C.C. with less current than the K.C.C. The case affords fresh light on the action of the lumbrical muscles. The common teaching on the action of these is that they, with the aid of the interossei, flex the metacarpo-phalangeal and extend the interphalangeal joints. Presumably this is based on their anatomical attachments and on the deformity (main-en-griffe) and disability that occurs if they are paralysed. Investigation of this patient leads me to the conclusion that the usual description omits a synergic action which is of paramount importance. Fig. 5 shows the typical main-en-griffe position of the fingers, and from that, although flexion can be performed, no further extension of the fingers is possible, *i.e.* the chief handicap is absent extension and, from current teaching, the inference is that this missing movement is usually carried out by the tiny lumbricals. Fig. 6 brings disillusionment, for it is only necessary to push the bases of the proximal phalanges forward passively and the patient becomes capable of full extension and escapes from the "gryphon's claw." This observation is confirmed in the natural hand as follows. If one flexes the index finger to a right angle at the metacarpo-phalangeal joint and, at the same time, attempts to straighten the last two joints of the finger, one becomes conscious of a sense of effort in the palm and of an unconsummated action. If, holding the one index finger thus, pressure is made firmly with the opposite forefinger on the back of the base of the proximal phalanx, a feeling of release from strain will be found, and extension is simultaneously easily and fully

PLATE IV.

FIG. 5.—Case of V.D. Healed wound of wrist and total medio-ulnar palsy of hand, three months after injury. Shows main-en-griffe and wasting.

FIG. 6.—Case of V.D. Although complete lumbrical palsy, good extension of fingers possible when bases of fingers pressed forwards as in photo.

performed. Thus the extensor tendon is shown to be responsible for extension, but the lumbrical, which is an antagonistic flexor, exerts a synergic action, in that, by pulling forward the first phalanx, it fixes that bone and gives the extensor tendon a fixed fulcrum (the head of the first phalanx) on which to raise and extend the middle phalanx, and only when this *is* fixed can one extend the last phalanx. This seems to indicate that the main action of the lumbricals is neither primarily flexion or extension of the phalanges, but is that of elastic fixer of the first phalanx and so a vital accessory synergic muscle to the extensor communis digitorum. This aspect of the mechanism of the lumbricals seems to merit more attention than it has received in the past, and it is intended to deal more fully with it in a later communication.

## Figures and Tables

**Figure f1:**
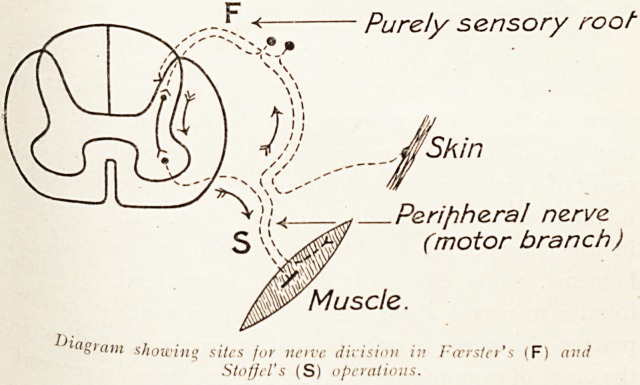


**Fig. 1. Fig. 2. f2:**
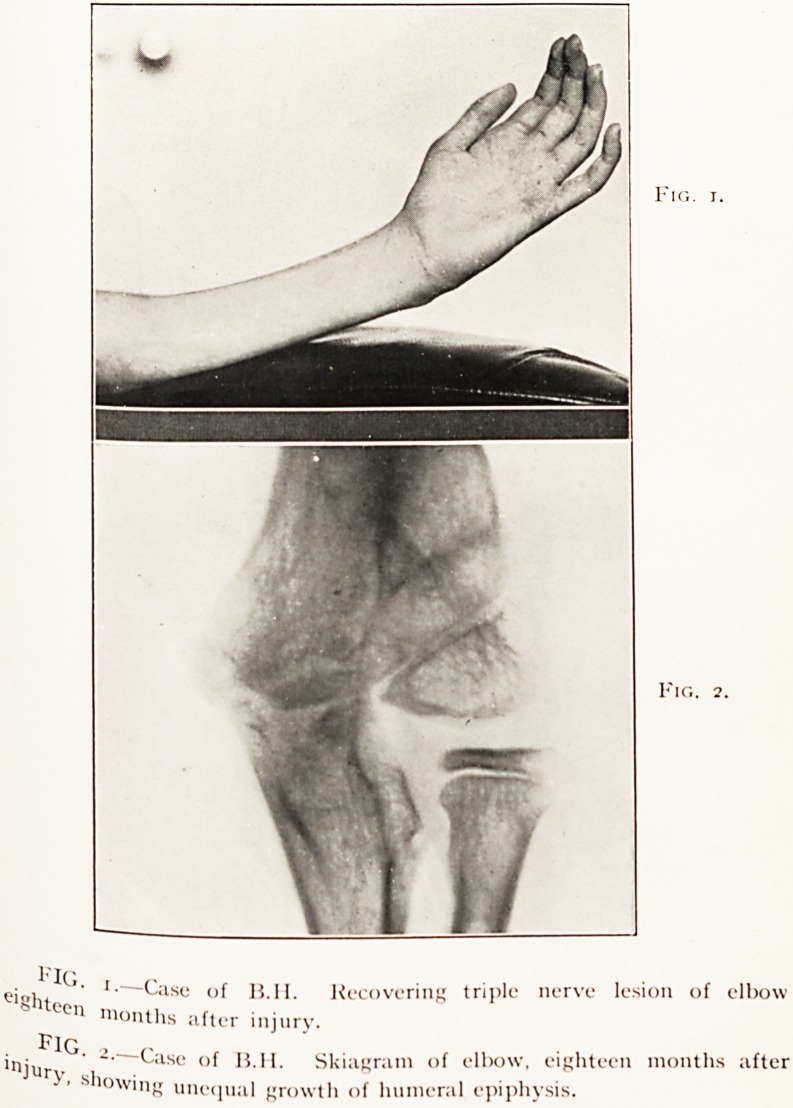


**Fig. 3. Fig. 4. f3:**
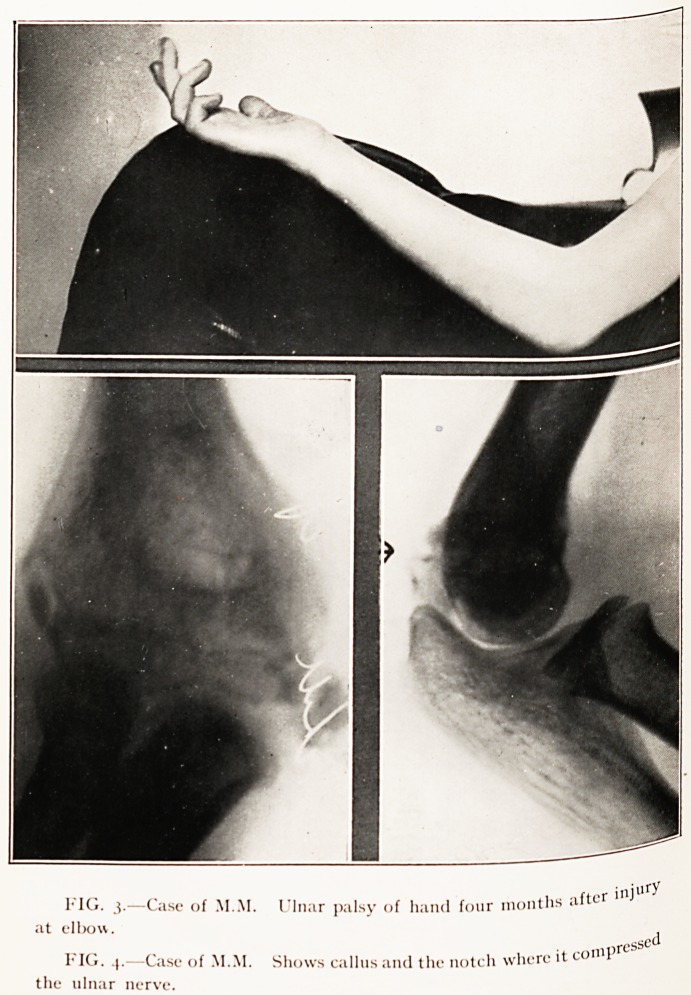


**Fig. 5. Fig. 6. f4:**